# P-940. Diagnostic Workup of Bacteremia Post TAVR, a Single Center Experience Over a Decade

**DOI:** 10.1093/ofid/ofae631.1131

**Published:** 2025-01-29

**Authors:** Kenneth Villareal, George (Trip) Zorn, Omar Almoghrabi, Peter Tadros, Goutham Marikanti, Yanming Li, Larry M Baddour, Wissam El Atrouni

**Affiliations:** University of New Mexico , Albuquerque, New Mexico; University of Kansas Medical Center, Kansas City, Kansas; University of Kansas Medical Center, Kansas City, Kansas; University of Kansas Medical Center, Kansas City, Kansas; University of Kansas Medical Center, Kansas City, Kansas; University of Kansas Medical Center, Kansas City, Kansas; Mayo Clinic College of Medicine, Rochester, MN; University of Kansas Medical Center, Kansas City, Kansas

## Abstract

**Background:**

Prosthetic valves are considered a risk factor for endocarditis however diagnosing prosthetic valve endocarditis (PVE) is complicated by acoustic shadowing on echocardiography. In 2023, the ISCVID published an update to the Duke Criteria (Duke 2023) which intended to increase the sensitivity in diagnosing PVE. The purpose of this study was to examine how TAVR infective endocarditis (TAVR-IE) was diagnosed at our institution

**Figure:**
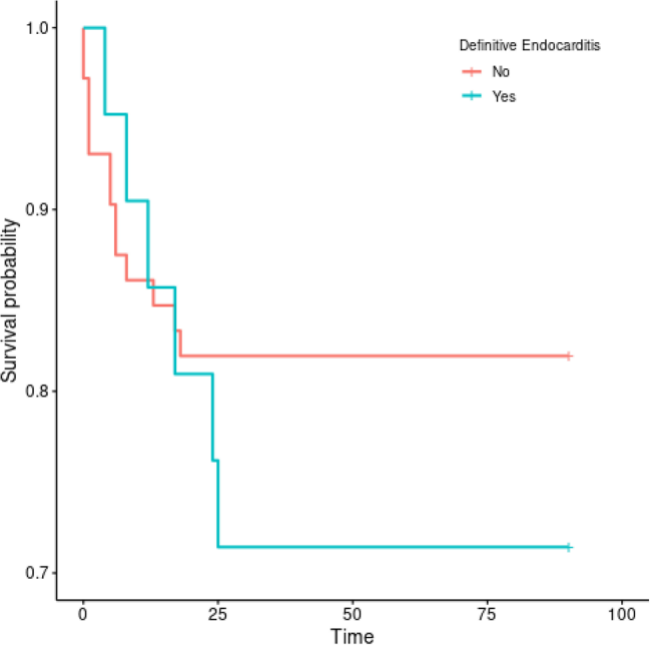

90 Day Mortality Kaplan Meier Curve

**Methods:**

HERON was utilized to match cases between ACC/STS databases and the EMR at The University of Kansas Health System (TUKHS). Patients with bacteremia occurring after a TAVR were reviewed. Each case was evaluated if they met rejected/possible/definitive endocarditis criteria in the Modified Duke Criteria (Duke 2005) and the Duke 2023 Criteria. To adjudicate the diagnosis, an expert panel reviewed all cases that met definitive endocarditis criteria in any edition of the Duke Criteria.

**Results:**

A total of 2371 TAVRs were conducted at TUKHS from 2012 to 2023. We identified 146 cases of post procedure bacteremia. We excluded 53 cases leaving 93 in the cohort. Transeophageal echocardiography was utilized in about half of the cases (TTE 73, TEE 45). F-FDG positron emission tomography/CT was done in six patients and Cardiac CT in one patient. Cultures returning with *Enterococcus* spp (16). viridans group streptococci (15), and *Staphylococcus* spp. (15) had similar incidence. About half had either atypical organisms (40) on 2005 Duke criteria or polymicrobial growth (7). Definitive criteria were met in 18 and 21 patients for the Duke 2005 and Duke 2023 Criteria respectively. Two cases had minor microbiological criteria upgraded to major in Duke 2023. One case was promoted to definitive endocarditis based on Cardiac CT. Upon adjudication the panel agreed with Duke 2023 Criteria classification. Sensitivity was calculated 85%/100% for Duke 2005 and Duke 2023 Criteria respectively. Kaplan Meier curves calculated no significant difference of 3 month mortality between TAVR patients who had met definitive criteria and those who did not. (Figure #1)

**Conclusion:**

The 2023 ISCVID Duke Criteria in this study appeared to have superior sensitivity for TAVR-IE when compared to the older Modified Duke Criteria published in 2005.

**Disclosures:**

**Larry M. Baddour, MD**, UpToDate, Inc.: Royalty payments (authorship duties). **Wissam El Atrouni, MD**, ViiV Healthcare: Advisor/Consultant

